# Direct Laser-Induced Breakdown Spectroscopy Analysis of Estuarine Suspended Particulate Matter Collected on Filters

**DOI:** 10.3390/molecules31040647

**Published:** 2026-02-13

**Authors:** Carlos Renato Menegatti, Mariany Sousa Cavalcante, Ricardo Schneider, Gustavo Pontes, Giorgio S. Senesi, Gustavo Nicolodelli

**Affiliations:** 1 School of Engineering of Lorena, University of São Paulo, Estrada Municipal do Campinho, Lorena 12602-810, SP, Brazil; renatomenegatti@usp.br; 2 Coastal Biogeochemistry Laboratory, Institute of Marine Sciences, Federal University of Ceará, Fortaleza 60455-970, CE, Brazil; cavalcante.mariany@gmail.com; 3 Polymers and Nanostructures Group, Federal University of Technology—Paraná (UTFPR), Toledo 85902-490, PR, Brazil; rikardos17@gmail.com; 4 Department of Physics, Federal University of Santa Catarina, Florianópolis 88040-900, SC, Brazil; gustavopontesfsc@gmail.com; 5 National Council of Research (CNR)—Istituto per la Scienza e Tecnologia dei Plasmi (ISTP)—Sede di Bari, 70126 Bari, Italy

**Keywords:** laser-induced breakdown spectroscopy, suspended particulate matter, estuarine system, metals, salinity

## Abstract

Estuaries are dynamic environments that influence the transport of metals and nutrients from land to sea, with suspended particulate matter (SPM) serving as a key vehicle for them. Laser-Induced Breakdown Spectroscopy (LIBS) offers a rapid, versatile, and non-destructive approach for multi-element analysis of SPM, allowing their direct measurement on collected filters without complex sample preparation. In this study, LIBS was applied to evaluate the spatio-temporal variability of major and trace elements (Si, Fe, Al, Ti, Na, Li, K, Rb, Ca, and Mg) along the Pacoti River estuary, Brazil, during rainy and dry seasons. Elemental patterns generally reflected the salinity gradient and tidal dynamics, highlighting element-specific behaviors with most elements showing inverse correlations with salinity, while Ca and Mg displayed positive correlations. These findings confirm the potential of LIBS as a powerful tool for environmental monitoring, providing rapid, high-throughput characterization of SPM and enabling an improved understanding of biogeochemical processes in estuarine systems.

## 1. Introduction

Estuaries are transitional environments that modulate the transfer of metals from the continent to the ocean [1]. The mixing of freshwater and seawater creates a strong physicochemical gradient along the estuarine channel, which significantly affects the mobility and fate of metals [2]. Additionally, the suspended particulate material (SPM) plays a significant role in the transport of heavy metals due to its high surface area and varied composition, including inorganic materials (e.g., clay minerals) and organic matter, which enables interaction with metals [1,3]. Changes in environmental conditions, such as salinity, pH, temperature, dissolved oxygen, and organic matter concentration, can influence the adsorption or desorption of metals from SPM [4]. In turn, SPM can settle on the estuarine bottom and sediment, acting as a sink for metals. Additionally, the bottom sediments can be resuspended, becoming a source of metals to the water column [5]. The balance of these processes generally favors the retention of metals in the estuary, yet considerable exportation occurs [6,7].

Laser-Induced Breakdown Spectroscopy (LIBS) [8] has emerged as a promising analytical tool in environmental applications, mainly due to its speed, versatility, and minimally destructive nature. The technique enables the identification and quantification of chemical elements directly in solid, liquid, or gaseous samples without the need for complex preparation, making it ideal for the in situ monitoring of soils, water, waste, and atmospheric pollutants. Furthermore, LIBS contributes to environmentally friendly analyses by minimizing the use of chemical reagents and reducing waste generation, thus supporting the implementation of sustainable practices in environmental monitoring and remediation.

In particular, LIBS has been scarcely explored for the analysis of environmental filters, despite its strong potential in this field. Some studies have demonstrated its feasibility in the characterization of atmospheric particulate matter collected on filters, enabling the rapid and multi-element detection of pollutants, heavy metals, and other relevant constituents [9,10]. Additionally, reports have shown its application in the investigation of biomass on filters containing algae, highlighting the capability of LIBS to discriminate between organic and inorganic components [11]. These examples emphasize the promising role of the technique in environmental monitoring, especially when applied to filter-based collection systems that concentrate chemical information in a cost-effective and easily handled format.

Given that SPM is typically collected on membrane filters for subsequent laboratory analysis, the ability of LIBS to perform direct, multi-element detection on these filters represents a significant advantage. Conventional techniques, such as inductively coupled plasma mass spectrometry (ICP-MS) and X-ray fluorescence (XRF), present distinct limitations when applied to the direct analysis of particulate materials. ICP-MS typically requires sample digestion or complex chemical preparation prior to analysis, which can alter the original composition of the sample, increase analytical time, and generate chemical waste [12]. On the other hand, XRF suffers from reduced sensitivity to low atomic number elements (Z < 11) due to the weak emission of characteristic X-rays and strong absorption by the sample matrix [13]. In contrast, LIBS can directly interrogate particulate matter on the filter surface without digestion or matrix alteration, thereby preserving the spatial distribution of particles and significantly reducing the analytical time [14]. This capability is particularly valuable for estuarine studies, where the rapid and accurate assessment of metal loads and nutrient dynamics is essential for understanding biogeochemical processes and evaluating anthropogenic impacts. By enabling high-throughput screening of SPM samples with minimal environmental footprint, LIBS emerges as a powerful approach to monitor the sources, transport, and fate of trace elements in transitional aquatic systems [11].

The present study aimed at utilizing LIBS as a tool for the evaluation of the spatio-temporal patterns and seasonal/tidal variability of metals and nutrients (Si, Fe, Al, Ti, Na, Li, K, Rb, Ca, and Mg) in the SPM of the Pacoti River estuary, NE Brazil. The river is moderately affected by dams that were built along it to mitigate the effects of water scarcity in the area [15]. The Pacoti River estuary is associated with a mangrove forest (1.44 km^2^) [16,17] that has been an environmental protected area since 2000. Although the estuary is part of the protected area, industrial and real estate activities have caused damage to this system due to the discharge of untreated sewage [18].

## 2. Results and Discussion

### 2.1. LIBS Spectra and Elemental Composition of SPM

**Figure 1:**
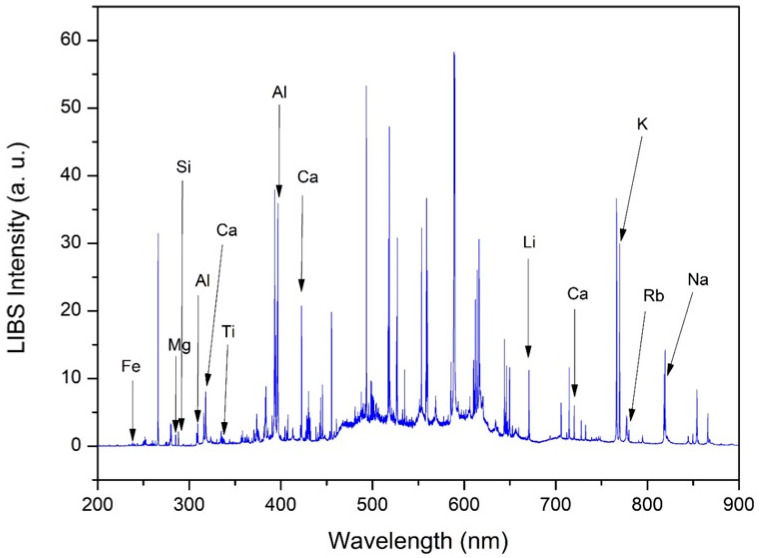
Full-range LIBS spectrum obtained from suspended particulate matter collected on membrane filters, shown to illustrate the presence of multiple elements in this complex matrix. The increased background in the 450–650 nm region is mainly due to line congestion and continuum emission under our experimental conditions.

Figure 1 presents a typical LIBS spectrum of a filter sample collected in April 2022 at site 1. The emission lines identified in the spectrum correspond to several elements present in the suspended particulate matter. In detail, the following emission lines were used: Fe II—238.20 nm; Mg I—285.21 nm; Si I—288.15 nm; Ti II—338.02 nm; Al I—394.40 nm; Ca I—422.67 nm; Li I—670.79 nm; K I—769.89 nm; Rb I—780.02 nm; and Na I—818.32 nm. The emission lines highlighted in Figure 1 were identified using the NIST Atomic Spectra Database [19] and are presented as representative examples of the elements detected in the LIBS spectra. These emission lines were chosen based on their relative intensity, signal-to-background ratio, and spectral isolation, ensuring reliable comparisons of LIBS emission signals across temporal and spatial datasets.

### 2.2. Temporal Variation of Salinity, SPM and LIBS Emission Signals in Estuarine Samples

**Figure 2:**
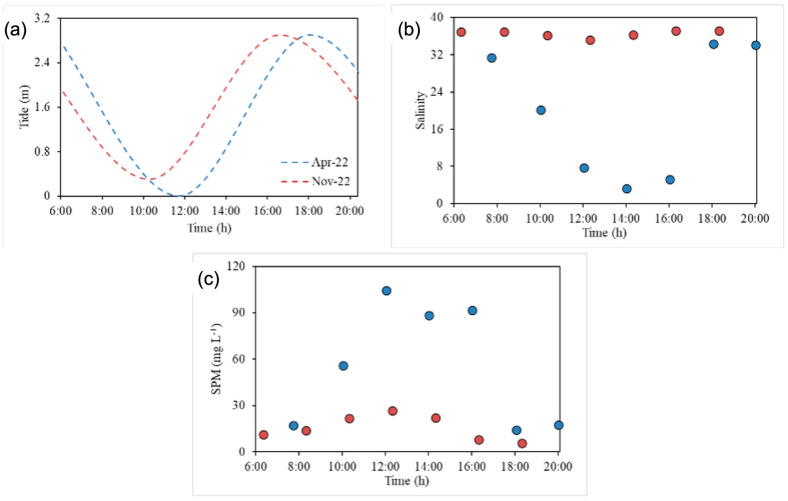
Tidal (**a**), salinity (**b**), and SPM variation (**c**) at a fixed point in the main channel of the Pacoti River estuary. Blue circles: rainy season (April 2022). Red circles: dry season (November 2022).

Temporal measurements of salinity were carried out on samples collected at station 2. The tide level varied from 0 to 3.0 m in the rainy season (April 2022) and from 0.3 to 2.9 m in the dry season (November 2022) (Figure 2a). The salinity behavior reflected tidal variation, decreasing during the ebb tide and increasing during the flood tide. In particular, salinity varied from 3.3 to 34.4 PSU in the rainy season, whereas its variation was narrower during the dry season, ranging from 35.2 to 37.1 PSU, due to the lower freshwater input and consequent increase in marine intrusion [20] (Figure 2b). The delay between salinity and tidal variation occurred because salt intrusion depends on advective and dispersive transport processes, which are slower than tidal wave propagation. Furthermore, the delay was affected by the location within the estuary, featuring an increase upstream [21].

During the rainy season, a period of high river inflow, SPM concentrations ranged from 14.2 to 104.5 mg‧L^−1^ with an average of 55.6 mg‧L^−1^. During the dry season, the estuarine system was heavily leached by seawater [22], and the SPM concentrations decreased to a range from 5.7 to 26.6 mg‧L^−1^, with an average of 15.6 mg‧L^−1^. In both seasons, the SPM levels tended to increase with the ebb tide and decrease with the flood tide (Figure 2c). The SPM concentrations and their seasonal patterns measured in this study were similar to those found in other semi-arid estuaries, and the same seasonal pattern was observed. For example, SPM levels in the lower Jaguaribe River estuary, located in the same semi-arid region, varied from 54.8 to 81.4 mg‧L^−1^ (74.8 mg‧L^−1^) during the rainy season and from 15.2 to 52.9 mg‧L^−1^ (29.6 mg‧L^−1^) during the dry season.

**Figure 3:**
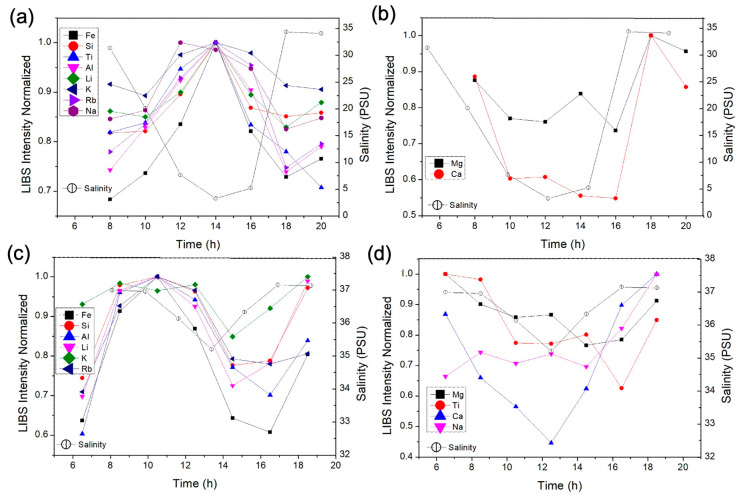
Normalized LIBS emission signals (background-subtracted integrals of selected lines) and salinity as a function of time at site 2 for two distinct seasonal periods: April 2022 (**a**,**b**), rainy season) and November 2022 (**c**,**d**), dry season). For each element, the most intense and interference-free line was chosen (e.g., Al at 394.40 nm and Ca at 422.67 nm). The signals were normalized to the maximum value observed for each element, allowing all elements to be plotted on a common scale and facilitating comparison of their temporal behavior.

Figure 3 presents the normalized LIBS emission signal associated with the element emission as a function of time for two distinct seasonal periods: April 2022 (Figure 3a,b), representing the rainy season, and November 2022 (Figure 3c,d), corresponding to the dry season. The signal of each element was normalized by the emission line area after background subtraction, allowing all elements to be plotted on the same scale and facilitating comparison of their temporal behavior.

In particular, Figure 3a,b illustrates the temporal behavior of the LIBS emission signals of two distinct elemental groups, i.e., Fe, Si, Ti, Al, Li, K, Rb, Na (a), and Ca, Mg (b), each exhibiting characteristic patterns in relation to salinity. The group of elements in Figure 3a displayed a negative correlation with salinity, with higher LIBS emission signals occurring under lower salinity conditions, whereas the group in Figure 3b showed a positive correlation, following the same trend as salinity fluctuations (Table 1). These results suggest that elemental concentrations are primarily controlled by continental inputs or resuspension processes that become more pronounced under low-salinity conditions (low tide). Normally, metals are inversely related to salinity due to a decrease in freshwater input, as rivers are the primary sources of particulate metals to estuaries [1,23]. SPM metals enter the rivers through rock and soil weathering, atmospheric deposition, and anthropogenic activities [24]. In contrast, Mg and Ca displayed a positive correlation with salinity, which is consistent with their predominantly marine origin, and is probably due to their adsorption as cations from seawater to SPM [25].

Figure 3c,d illustrates, respectively, the LIBS emission signals patterns of the elements Fe, Si, Al, Li, K, and Rb, and Mg, Ti, Ca, and Na during the dry season. The group in Figure 3c exhibited a relatively consistent negative correlation to salinity and SPM (Table 1), whereas the group in Figure 3d did not display a defined or consistent pattern, except for Ca, which was positively correlated with salinity and SPM (Table 1). These results suggest the relevance of SPM in elemental transport from land to ocean, and that seasonal changes would control SPM metals in the Pacoti River estuary. Lacerda et al. [7] showed that pollutant reactivity (e.g., mercury) increased in a semi-arid estuary during the dry season due to the prolonged water residence time, i.e., the oceanic force was larger than the freshwater flux in the estuary, trapping pollutants within SPM. A similar mechanism might have occurred in the Pacoti River estuary, favoring biogeochemical reactions and altering the chemical partitioning of SPM metals in the water column.

To assess the reproducibility of the LIBS measurements, average standard deviations (expressed as percentages) were calculated and are presented in Table S1 in the Supplementary Materials. The highest mean standard deviation was observed for Na during the temporal sampling of November 2022, reaching 14%. In contrast, the lowest mean standard deviation was obtained for Rb during the temporal sampling of April 2022, with a value of 2.9%. These results indicate that although variability exists among elements and sampling periods, the overall precision of the LIBS measurements remained within acceptable limits for environmental monitoring applications.

### 2.3. LIBS Emission Signals, Salinity and SPM Profiles Across Estuarine Sampling Sites in the Rainy and Dry Seasons

**Figure 4:**
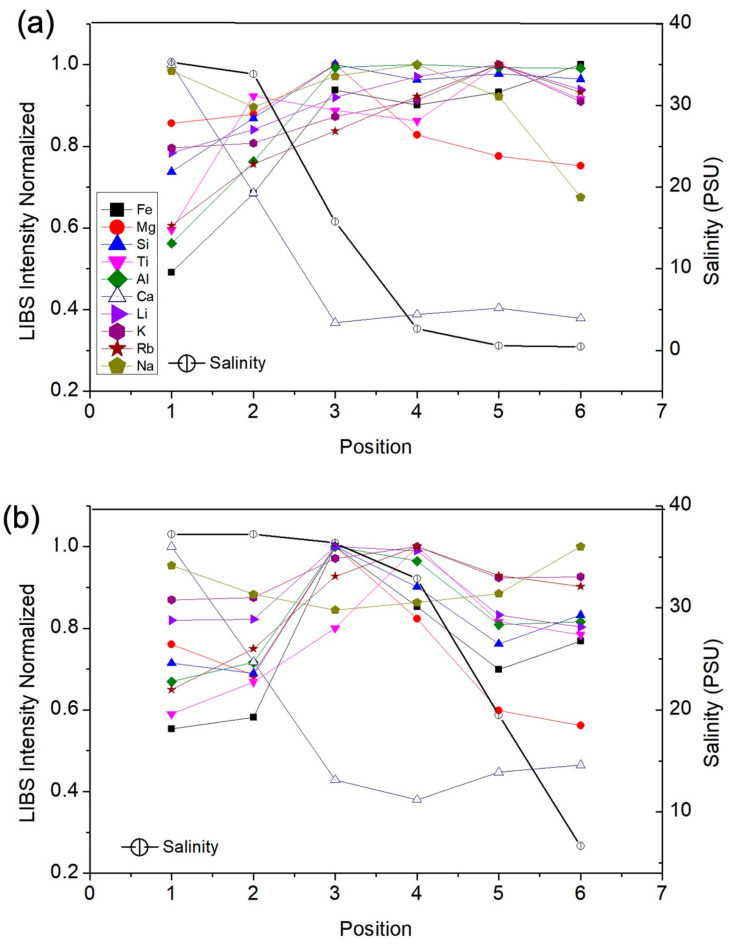
Spatial variation of normalized LIBS emission signals (background-subtracted integrals of selected lines) and salinity across sampling sites in the Pacoti River estuary during April 2022 (**a**), rainy season) and November 2022 (**b**), dry season). For each element, the most intense and interference-free line was chosen (e.g., Al at 394.40 nm and Ca at 422.67 nm). The emission signals were normalized to the maximum value observed for each element, allowing all elements to be plotted on a common scale and facilitating a comparison of their spatial distribution along the estuary.

Figure 4 presents the normalized LIBS emission signals measured by LIBS in samples collected from stations 1 (downstream) to 6 (upstream) as a function of sampling location for two distinct seasonal periods, i.e., March 2022 (rainy season) and November 2022 (dry season), which are shown, respectively, in Figure 4a,b. In Figure 4a, the LIBS emission signals of most elements exhibited a consistent pattern inversely related to salinity, except calcium (Ca), which displayed a distinct behavior positively correlated with salinity. Similarly, the LIBS emission signals of the elements in Figure 4b followed a characteristic distribution pattern, again with Ca differing from the general trend due to its apparent correlation with salinity.

The positive correlation observed for Ca and Mg with salinity can also be attributed to their strong association with marine-derived particles and carbonates, as well as their tendency to adsorb onto suspended particulate matter under increasing ionic strength. In contrast, sodium, despite its high abundance in seawater, is predominantly present in the dissolved phase and exhibits weak retention in particulate material. Consequently, Na does not necessarily follow the same spatial trend as Ca and Mg in suspended particles, resulting in a comparatively constant or even anticorrelated behavior along the estuary.

**Figure 5:**
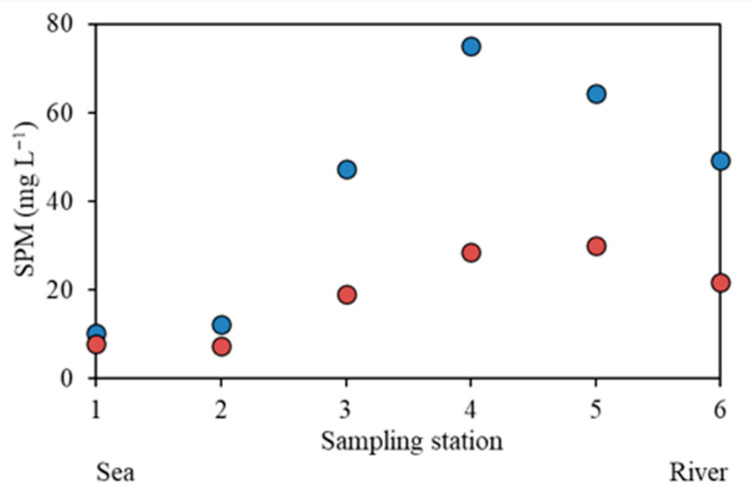
Spatial variation of SPM along the sampling stations in the main channel of the Pacoti River estuary. Blue circles: Rainy season (April 2022). Red circles: Dry season (November 2022).

As expected, due to the progressive influence of freshwater inputs, salinity decreased consistently from station 1 to station 6 in both the rainy (Figure 4a) and dry seasons (Figure 4b). Similarly, SPM concentrations displayed a decreasing pattern downstream due to seawater dilution during estuarine mixing [22] (Figure 5). In particular, the highest SPM levels were measured at stations 4 and 5 during the rainy and dry seasons, possibly due to processes such as resuspension of the bottom, erosion, or changes in sources.

Most SPM elements (Fe, Si, Al, Ti, Li, K, and Rb) exhibited a decrease in LIBS emission signals from upstream to downstream stations during both rainy and dry seasons, possibly caused by seawater dilution and particle settling [23], with an inverse relationship with salinity. In particular, Al, Fe, and Si showed an abrupt decrease passing from stations 3 to 1. Furthermore, in the dry season, the elements Fe, Si, Al, Ti, Li, K, and Rb presented the maximum LIBS emission signals at intermediate stations 3 and 4, likely reflecting localized accumulation or resuspension processes.

In contrast, Ca exhibited a marked increase along the estuarine gradient during both seasons, indicating a positive relationship with salinity and reflecting its predominantly marine influence in the particulate fraction. Magnesium showed a different behavior, with LIBS emission signals increasing up to station 3 and decreasing toward the downstream region, suggesting the combined influence of freshwater inputs, mixing processes, and particle–water interactions. Unlike the other elements, Na displayed relatively constant values along the estuary, indicating that its particulate-associated signal was not directly coupled to the salinity gradient. This behavior is consistent with ion-exchange and desorption processes that may limit the retention of Na in suspended particulate matter as salinity increases. Overall, these results highlight the differential influence of estuarine hydrodynamics, freshwater contributions, and seasonal conditions on the distribution of major and trace elements associated with estuarine SPM.

Although a direct comparison with reference techniques such as ICP-MS would be valuable for absolute quantification, the present study primarily aimed to evaluate the capability of LIBS to provide rapid, field-deployable, and qualitative assessment of emission signal variations in estuarine suspended particulate matter. The consistent associations observed with salinity gradients and tidal influence demonstrate that LIBS captures reproducible relative changes under field conditions. Nevertheless, because the spatial sampling represents snapshot conditions, site-to-site differences should be interpreted cautiously and regarded as indicative rather than strictly attributable to intrinsic spatial variability. Future studies integrating LIBS with reference analytical techniques may further support quantitative validation.

## 3. Materials and Methods

### 3.1. Study Area

**Figure 6:**
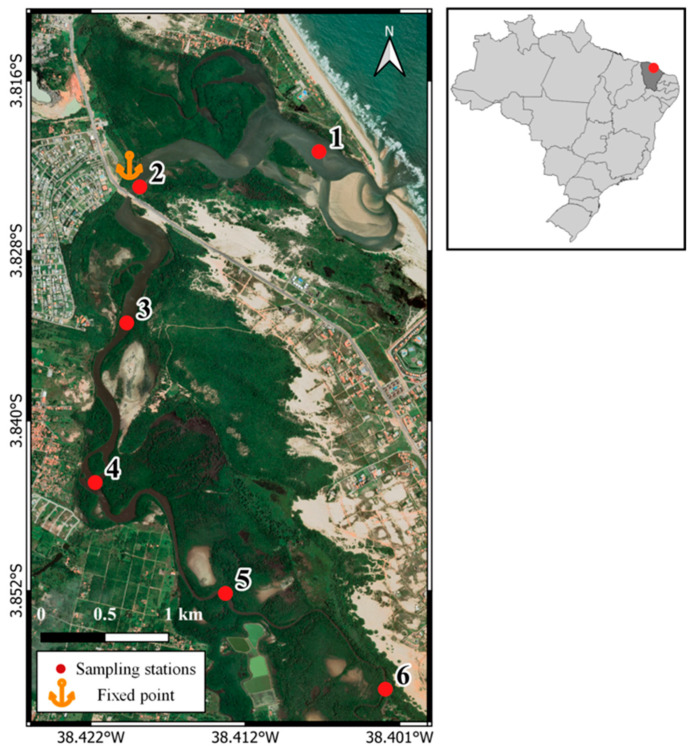
Location of the study area and the sampling stations in the Pacoti River estuary, NE Brazil.

The Pacoti River estuary is located in the Brazilian equatorial continental shelf (Figure 6) and features a semi-arid climate characterized by a low precipitation rate (<700 mm year^−1^), concentrated rainfall events (4–5 months), and an evaporation rate (1900 mm year^−1^) higher than the precipitation rate during most of the year. The watershed covers an area of roughly 1000 km^2^, with a length of 150 km [26].

The semidiurnal tidal cycle is characterized by two high and two low tides of approximately equal size every lunar day, and a mesotidal range with a maximum amplitude of 3.3 m [20,27]. In semi-arid estuaries, it is necessary to consider the combined effect of vectors with different directions, i.e., the fluvial inflow to the estuary and tidal forcing. The wave propagation curve in the estuary was estimated using daily water level data provided by the Brazilian Hydrography and Navigation Department for the Port of Mucuripe (CE).

### 3.2. Sampling Strategy

Sampling was performed in two ways: spatial (at six sampling stations along the principal channel) and diel (at a fixed point in the main channel for 13 h at intervals of 2 two hours). Spatial and diel samplings were carried out in April 2022 (rainy season) and November 2022 (dry season). The diel sampling was conducted at site 2 because of its location in the lower estuary, a transition zone between fluvial and marine waters. The lower estuary is characterized by strong tidal influence and pronounced gradients in salinity and suspended particulate matter, making it particularly suitable for evaluating tidal-driven variability in SPM composition.

Subsurface water salinity was measured using a SontekCast Away CTD. The temperature (T) and pH were measured in situ using a multiparameter probe (Hanna Instruments, HI-9828, Smithfield, RI, USA). A total of 28 subsurface water (0.5 m) samples were collected in an acrylic Van Dorn bottle. It is important to note that the spatial interpretation presented here is based on snapshot sampling and does not aim to resolve the full temporal variability associated with tidal cycles.

Water temperature and pH were monitored during sampling; however, no significant variations were observed among sites and seasons. As a result, these parameters were not considered as primary drivers of the LIBS signal variability and are not further discussed.

Temporal sampling at different tidal stages was performed only at site 2, which was selected as a representative mixing zone within the estuary. At the remaining sites, samples were collected under single tidal conditions and are therefore interpreted as spatial snapshots rather than fully resolved tidal signals. This approach allows for an exploratory assessment of spatial variability while acknowledging the limitations imposed by tidal dynamics in estuarine systems.

### 3.3. Laboratory Measurements

Suspended particles were collected from water samples filtered immediately after sampling through pre-weighed and pre-combusted (at 450 °C, 12 h) Whatman glass microfiber filters with a 0.7 µm mesh. Each sample was slowly added to a Millipore glass filtration apparatus to ensure maximum particle concentration in the filter without clogging it. The vacuum pressure was then monitored to ensure that it did not exceed 400 mmHg. Filtration volumes ranged from 100 to 1600 mL, depending on the particle concentration. Subsequently, filters were desalinated with Milli-Q water and then dried at 75 °C for 1 h in an oven for further SPM and particulate metal measurements.

For SPM quantification, the filters, with a diameter of 47 mm, were weighed on an analytical balance. The SPM concentration was calculated by subtracting the weight of the filters after filtration from the weight before filtration, and then dividing by the exact filtered volume of each sample, expressed in mg‧L^−1^. The SPM measurements were made in duplicate at each sampling station and data were used as their averages. More details about the procedure are described in Neukermans et al. [28].

### 3.4. LIBS Measurements

LIBS measurements were carried out using an Applied Spectra J200 system equipped with a Q-switched Nd:YAG laser operating at 266 nm, with a pulse duration of approximately 6 ns and a repetition rate of 10 Hz. According to the manufacturer’s specifications, the laser beam presents a multimode spatial profile. The pulse energy delivered to the sample surface was 15 mJ. The laser beam was focused onto the sample surface using the standard focusing optics provided by the manufacturer. Based on the Applied Spectra J200 specifications, optimal focal adjustment results in a nominal laser spot diameter of approximately 150 µm at the sample surface. This value corresponds to the estimated beam waist at the focus, assuming proper focal alignment, and was not determined from direct measurements of the ablation crater.

Plasma emission was collected in a side-view geometry using a fiber-optic collection system positioned at 30° relative to the incident laser beam and coupled to a multichannel charge-coupled device (CCD)-based spectrometer (Avantes B.V., Apeldoorn, The Netherlands) with a spectral range from 186 to 1042 nm. In this study, a fixed delay time of 0.7 µs and an integration time of 1 ms were applied to all measurements and spectral channels. This configuration was selected to reduce continuum background emission while maintaining sufficient signal intensity for line detection. The same acquisition conditions were applied to all samples to ensure measurement consistency and reproducibility.

LIBS measurements were performed in a continuous line-scan mode, in which the laser was fired continuously while the sample was translated. This acquisition strategy prevented multiple laser shots from being delivered to the same surface location, thereby avoiding crater overlap. The distance between consecutive laser shots was not directly measured. Sample positioning and translation were performed using a motorized XY stage with 100 mm × 100 mm travel (0.2 µm resolution) and a Z stage with 35 mm travel (1 µm resolution), integrated into the LIBS system.

For each filter sample, a total of 1820 LIBS spectra were acquired along 20 parallel lines of 1 cm length. Along each line, 91 laser shots were accumulated. The large number of spectra collected over multiple spatial locations were used to minimize the influence of potential microscale heterogeneities. At the scale of the LIBS sampling area, the samples were considered macroscopically homogeneous. For each sample, all spectra were averaged, significantly reducing shot-to-shot fluctuations and improving measurement repeatability. This approach ensures robust relative comparisons among sampling sites and seasons, which as the primary objective of the present study.

Blank filter samples were analyzed under the same LIBS conditions to assess potential contributions from the filter substrate. Although emission lines associated with elements intrinsic to the filter material were detected, their intensities were consistent across measurements and did not exhibit qualitative variations or correlations with salinity. Therefore, these contributions were treated as constant background signals and were not included in the spectral interpretation presented in this study.

## 4. Conclusions

This study demonstrated the effectiveness of LIBS as a rapid and performant analytical technique for assessing suspended and particulate metals in filtered estuarine samples. The technique proved valuable not only for detecting the presence and variability of metallic elements but also for guiding the selection of samples for more detailed analyses.

Spatial patterns revealed that the elements Fe, K, Li, Al, Ti, Rb, and Si decreased downstream during both the rainy and dry seasons, suggesting a predominantly fluvial origin. In contrast, Ca and Mg LIBS emission signals increased toward the estuary mouth, while Na remained relatively constant. Temporal analysis showed that Fe, Si, Al, Li, K, and Rb LIBS emission signals increased during ebb tide, coinciding with lower salinity, thus indicating riverine input. Conversely, Ca and Mg exhibited the opposite behavior, with higher LIBS emission signals during flood tide and elevated salinity, suggesting marine influence and/or estuarine mixing processes. Ti and Na displayed season-dependent trends, decreasing with salinity during the rainy season and increasing during the dry season.

The inverse correlation between most SPM metals and salinity confirmed the role of the river as the primary source of these elements. However, the positive correlation with salinity observed for certain metals suggested adsorption onto SPM during estuarine mixing and/or potential marine contributions. Overall, SPM emerged as a key factor in regulating the distribution and transport of metals within the Pacoti River estuary.

## Figures and Tables

**Table 1:** Pearson correlation coefficients between LIBS-derived emission signals and two environmental variables: salinity and suspended particulate matter (SPM). Temp. Samp. refers to temporal sampling, specifically the seven consecutive time periods monitored at site 2 during April (rainy season) and November (dry season). Spat. Samp. refers to spatial sampling, corresponding to measurements performed across the six sampling stations along the Pacoti River estuary.

Elem.	April 2022 Temp. Samp.		November 2022 Temp. Samp.		April 2022 Spat. Samp.		November 2022 Spat. Samp.	
	Sal.	SPM	Sal.	SPM	Sal.	SPM	Sal.	SPM
Fe	−0.79	0.72	−0.42	0.39	−0.90	0.83	0.07	0.56
Mg	0.81	−0.83	0.22	−0.31	0.49	−0.29	−0.72	−0.11
Si	−0.67	0.59	−0.33	0.22	−0.81	0.81	0.00	0.49
Ti	−0.82	0.79	0.24	−0.13	−0.61	0.55	0.22	0.86
Al	−0.95	0.92	−0.52	0.50	−0.89	0.88	0.00	0.64
Li	−0.73	0.63	−0.21	0.08	−0.95	0.95	−0.44	0.37
Ca	0.94	−0.94	0.83	−0.94	0.83	−0.82	−0.46	−0.83
K	−0.87	0.83	−0.04	−0.20	−0.91	0.87	0.07	0.75
Rb	−0.98	0.93	−0.65	0.62	−0.94	0.88	0.39	0.90
Na	−0.94	0.95	0.39	−0.63	0.35	−0.01	0.62	−0.22

## Data Availability

All data generated or analyzed during this study are included in this published article.

## Supplementary Materials

The following supporting information can be downloaded at: https://www.mdpi.com/article/10.3390/molecules31040647/s1, Table S1: Average standard deviation (expressed as percentage) of LIBS-derived elemental signals. Temp. Samp. refers to temporal sampling, specifically the seven consecutive time periods monitored at site 2 during April (rainy season) and November (dry season). Spat. Samp. refers to spatial sampling, corresponding to measurements performed across the six sampling stations along the Pacoti River estuary.

## References

[1] Harmesa H., Wahyudi A.J., Wong K.H., Ikhsani I.Y. (2024). The Behaviour of Particulate Trace Metals in Marine Systems: A Review. Mar. Environ. Res..

[2] Barletta M., Lima A.R.A., Costa M.F. (2019). Distribution, Sources and Consequences of Nutrients, Persistent Organic Pollutants, Metals and Microplastics in South American Estuaries. Sci. Total Environ..

[3] Feng C., Guo X., Yin S., Tian C., Li Y., Shen Z. (2017). Heavy Metal Partitioning of Suspended Particulate Matter—Water and Sediment—Water in the Yangtze Estuary. Chemosphere.

[4] Santos T.T.L., Mounier J.L.S., Marins R.V. (2024). Trace Metal Partitioning in the Parnaíba Delta in Dry Season, Equatorial Coast of Brazil. Environ. Pollut..

[5] Oliveira R.C.B.d., Marins R.V. (2011). Trace Metals Dynamics in Soil and Estuarine Sediment as a Major Factor Controlling Contaminants Contribution to the Aquatic Environment: Review. Rev. Virtual Quim..

[6] Marins R.V., Lacerda L.D., Boas R.C.V. (1999). Relative Importance of Non-Point Sources of Mercury to an Industrialized Coastal System, Sepetiba Bay, SE Brazil. Mercury Contaminated Sites.

[7] Lacerda L.D.d., Marins R.V., Dias F.J.d.S. (2020). An Arctic Paradox: Response of Fluvial Hg Inputs and Bioavailability to Global Climate Change in an Extreme Coastal Environment. Front. Earth Sci..

[8] Fortes F.J., Moros J., Lucena P., Cabalín L.M., Laserna J.J. (2013). Laser-Induced Breakdown Spectroscopy. Anal Chem..

[9] Gonçalves D.A., Senesi G.S., Nicolodelli G. (2021). Laser-Induced Breakdown Spectroscopy Applied to Environmental Systems and Their Potential Contaminants. An Overview of Advances Achieved in the Last Few Years. Trends Environ. Anal. Chem..

[10] Senesi G.S., Harmon R.S., Hark R.R. (2021). Field-Portable and Handheld Laser-Induced Breakdown Spectroscopy: Historical Review, Current Status and Future Prospects. Spectrochim. Acta Part B At. Spectrosc..

[11] Hrdlička A., Horská J., Hegrová J., Bucková M., Prochazka D., Buday J., Pořízka P., Kanický V., Kaiser J. (2025). Influence of Sample Matrix and Filter Fixation on LIBS Signal in Analysis of Algae on Filter. J. Anal. At. Spectrom..

[12] Hou X., Jones B.T. (2000). Inductively Coupled Plasma/Optical Emission Spectrometry. Encycl. Anal. Chem..

[13] Jenkins R. (2012). X-Ray Fluorescence Spectrometry.

[14] Cremers D.A., Radziemski L.J. (2013). Handbook of Laser-Induced Breakdown Spectroscopy.

[15] Schettini C.A.F., Valle-Levinson A., Truccolo E.C. (2017). Circulation and Transport in Short, Low-Inflow Estuaries under Anthropogenic Stresses. Reg. Stud. Mar. Sci..

[16] Lacerda L.D.d., Menezes M.O.T.d., Molisani M.M. (2007). Changes in Mangrove Extension at the Pacoti River Estuary, CE, NE Brazil Due to Regional Environmental Changes between 1958 and 2004. Biota Neotrop..

[17] Marins R.V., Lacerda L.D., Araújo I.C.S., Fonseca L.V., Silva F.A.T.F. (2020). Phosphorus and Suspended Matter Retention in Mangroves Affected by Shrimp Farm Effluents in NE Brazil. An. Acad. Bras. Cienc..

[18] Soares M.O., Campos C.C., Carneiro P.B.M., Barroso H.S., Marins R.V., Teixeira C.E.P., Menezes M.O.B., Pinheiro L.S., Viana M.B., Feitosa C.V. (2021). Challenges and Perspectives for the Brazilian Semi-Arid Coast under Global Environmental Changes. Perspect. Ecol. Conserv..

[19] (2025). NIST Database. https://physics.nist.gov/PhysRefData/ASD/LIBS/libs-form.html.

[20] Frota F.F., Truccolo E.C., Schettini C.A.F. (2016). Tidal and Sub-Tidal Sea Level Variability at the Northern Shelf of the Brazilian Northeast Region. An. Acad. Bras. Cienc..

[21] Dijkstra Y.M. (2024). Estuarine Adjustment: Dependence of Salinity Delay on the Forcing Timescale and Magnitude. J. Geophys. Res. Ocean..

[22] Dias F.J.d.S., Castro B.M., Lacerda L.D., Miranda L.B., Marins R.V. (2016). Physical Characteristics and Discharges of Suspended Particulate Matter at the Continent-Ocean Interface in an Estuary Located in a Semiarid Region in Northeastern Brazil. Estuar. Coast. Shelf Sci..

[23] Wang Y., Liu R., Zhang Y., Cui X., Tang A., Zhang L. (2016). Transport of Heavy Metals in the Huanghe River Estuary, China. Environ. Earth Sci..

[24] Gaillardet J., Viers J., Dupré B. (2014). Trace Elements in River Waters. Treatise on Geochemistry.

[25] Yao Q., Wang X., Jian H., Chen H., Yu Z. (2016). Behavior of Suspended Particles in the Changjiang Estuary: Size Distribution and Trace Metal Contamination. Mar. Pollut. Bull..

[26] Molisani M.M., Cruz A.L.V., Maia L.P. (2017). Estimativa da Descarga Fluvial Para Os Estuários Do Estado Do Ceará, Brasil. Arq. Cienc. Mar..

[27] Dias F.J.S., Marins R.V., Maia L.P. (2009). Hydrology of a Well-Mixed Estuary at the Semi-Arid NE Brazilian Coast. Acta Limnol. Bras..

[28] Neukermans G., Ruddick K., Loisel H., Roose P. (2012). Optimization and Quality Control of Suspended Particulate Matter Concentration Measurement Using Turbidity Measurements. Limnol. Oceanogr. Methods.

