# Supplementary material for: Direct Laser-Induced Breakdown Spectroscopy Analysis of Estuarine Suspended Particulate Matter Collected on Filters
Source: Molecules. 2026 Feb 13;31(4):647. doi: 10.3390/molecules31040647 (PMC12943375; doi:10.3390/molecules31040647)
Supplement: Supplementary file 1 [file molecules-31-00647-s001.zip › molecules-4077779-supplementary.pdf]

Table S1. Average standard deviation (expressed as percentage) of LIBS-derived elemental signals. Temp. Samp. refers to temporal sampling, specifically the seven consecutive time periods monitored at site 2 during April (rainy season) and November (dry season). Spat. Samp. refers to spatial sampling, corresponding to measurements performed across the six sampling stations along the Pacoti River estuary.

Elem.	Ap. 2022 Temp. Samp.	Nov. 2022 Temp. Samp.	Ap. 2022 Spat. Samp.	Nov. 2022 Spat. Samp.
Fe	9.1	8.3	7.0	7.3
Mg	6.7	6.3	5.2	5.9
Si	8.6	8.2	7.0	7.7
Ti	4.0	8.0	5.4	9.0
Al	6.8	5.5	5.5	5.9
Li	5.2	4.9	4.2	4.8
Ca	6.4	5.8	6.1	7.5
K	3.0	4.2	4.2	5.5
Rb	2.9	4.1	4.3	5.5
Na	9.9	14.0	9.3	11.4
